# Effectiveness of Cognitive Behavioral Couples Therapy on Increasing the Happiness and Marital Satisfaction of Infertile Couples: A Randomized Controlled Trial

**DOI:** 10.1002/hsr2.73369

**Published:** 2026-10-08

**Authors:** Malihe Gholamzade Deharab, Jamile Shahverdi, Elham Goodarzi, Azam Ardeh, Seyed Mojtaba Ahmadi

**Affiliations:** ^1^ CEO of Abarkoh Life Station Psychology Institute Shahid Sadoughi University of Medical Sciences Yazd Iran; ^2^ Department of Clinical Psychology, School of Behavioral Sciences and Mental Health Iran University of Medical Sciences Tehran Iran; ^3^ Social Determinants of Health Research Center Lorestan University of Medical Sciences Khorramabad Iran; ^4^ Department of Clinical Psychology, School of Public Health Shahid Sadoughi University of Medical Sciences Yazd Iran; ^5^ Department of Clinical Psychology, School of Medicine Shahid Beheshti University of Medical Sciences Tehran Iran

**Keywords:** cognitive behavioral couple therapy, happiness, infertility, marital satisfaction

## Abstract

**Background and Aims:**

Infertility is associated with several psychological problems. Considering the increasing prevalence of infertility and the role of psychological factors, the purpose of this study was to investigate the effectiveness of cognitive behavioral couple therapy on increasing the happiness and marital satisfaction among infertile couples.

**Methods:**

In this randomized controlled trial, 24 infertile couples were selected via convenience sampling from Infertility Center Kermanshah and randomly assigned to cognitive‑behavioral couple therapy (CBCT; 12 couples) or control (routine care; 12 couples). The CBCT group received ten 120‑minute weekly sessions. Data were collected using the Oxford Happiness and ENRICH Marital Satisfaction scales at pre‑test, post‑test, and two‑month follow‑up. Group homogeneity at baseline was assessed using Chi‑square and independent t‑tests. repeated‑measures ANOVA was conducted to evaluate main and interaction effects of group and time on the outcomes. All analyses were performed using SPSS‑25, with significance set at *p* < 0.05.

**Results:**

Repeated‑measures ANOVA revealed significant time × group interaction effects for happiness (F (2,86) = 13.48, *p* < 0.001, η^2^ = 0.239) and marital satisfaction (F(2,86) = 16.36, *p* < 0.001, η^2^ = 0.276). The CBCT group demonstrated significant improvements in both outcomes from pre‑test to post‑test and follow‑up, while the control group remained stable.

**Conclusions:**

Cognitive Behavioral Couple Therapy can be used in women and in particular infertile men to increasing the happiness and marital satisfaction among them.

## 1 Introduction

Most couples tend to have a child as they start sharing a common life together and infertility confronts them with crises. Despite the continuous progress being made in infertility treatment, infertility remains a substantial source of stress for couples experiencing it [1]. Infertility causes major crises that can be a threat to infertile couples' mental health. Accordingly, disorders in couples' relationships, helplessness, and other mental disorders are prevalent among these couples [2]. By definition, infertility is defined as 1 year of sexual activity without the use of contraceptives or birth control methods [3]. Infertility occurs for four reasons: male factor (40%), female factor (40%), mixed factors (both male and female, 10%), and unknown causes (10%) [2]. Overall rate of the infertility in Iran has been reported to be 2.1%. Besides, the overall prevalence of primary infertility and of secondary infertility in Iran were 5.2 and 3.2, respectively [4]. Infertile couples believe that this is a challenging experience in their lives leading to the various problems in their marital life [1].

Infertility is one of the personal‐ social problems that can expose affected people to multiple psychological pressures. It is a process that affects personality and mentality and has adverse effects on individual's emotions and communication. People with infertility often experience wide range of psychological disorders [5]. Research has shown that infertility makes people more susceptible to depression and stress, and this level is higher in women than in men [3].

The couple's problems include concepts such as conflict, dissatisfaction, and incompatibility. The concept of dissatisfaction is related to the conflict; that is, the increase of couple's conflicts will also increase their dissatisfaction and incompatibility [6]. Marital satisfaction is one of the important factors affecting the health of women. Marital satisfaction involves a set of factors including successfully solving the conflict, or engaging activities associated with happiness in marriage process. Marital satisfaction is defined as a process acquired during a common life, which requires adaptation of tastes and recognition of personality traits, creation of behavioral rules, and formation of communicative patterns [2]. Also, the marital satisfaction is an objective sentiment of pleasure, joy, and satisfaction experienced by a husband and wife [7].

Likewise, happiness is one of the most essential needs of human beings which refers to an individual's positive assessment of his/her life. Happiness is people's emotional and cognitive evaluation of their lives. The components of happiness are the satisfaction of life and the existence of positive moods and emotions [8]. Happiness is defined as being good, setting goods, and living well [9]. One of the factors that affects happiness is the individual's fertility or infertility rate [10].

There are several approaches to couple therapy that have affected different aspects of treatment. One of these approaches focuses on behavioral therapy. It is based on the treatment methods of solving couple's communication problems [11]. In another approach, therapists have been working to educate the couples about the correct patterns of thinking and effective communication by focusing on cognitive and behavioral therapy among them [6]. Additionally, married couples having disagreements in their marital life and facing difficulty in solving their problems need help from experienced counselors. This approach provides these counselors with practical guidance in order to teach the right communication skills to the couples and help them resolve their disputes [12]. The findings of Sudani's study indicated that couples who received behavioral couple therapy exhibited a significant reduction in conflict levels post‑treatment, compared to the control group [13].

Although previous studies have demonstrated the efficacy of cognitive‑behavioral couple therapy (CBCT) in reducing marital conflicts and alleviating psychological distress among infertile couples, most of this evidence comes from Western populations and has primarily focused on symptom reduction (e.g., depression, anxiety, or conflict). Moreover, few interventions have simultaneously targeted two positive psychological outcomes—happiness and marital satisfaction—as primary endpoints in the same infertile sample.

Existing local studies have either examined general couples without fertility issues or have used non‑cognitive‑behavioral approaches. Therefore, this study is unique in three ways: (1) it specifically targets infertile couples seeking treatment in an Iranian clinical setting, (2) it applies a structured CBCT protocol that integrates both cognitive restructuring and communication‑skill training, and (3) it evaluates both happiness and marital satisfaction as co‑primary outcomes, providing a more comprehensive picture of positive marital functioning.

As a result, the findings of this study may help health care providers provide mental support required for this group of people and implement appropriate programs to prevent infertility complications. So, this study aimed at investigating the effectiveness of the cognitive behavioral couple therapy on increasing the happiness and marital satisfaction among the infertile couples. We hope that this study will be a step towards advancing the treatment of infertility and reducing psychological problems in infertile couples.

## 2 Materials and Methods

### 2.1 Study Design and Setting

This study was a two‐arm, parallel‐group, randomized controlled clinical trial with a pre‐test, post‐test, and 2‐month follow‐up design. This trial was conducted in the spring of 2026 at Motazedi Infertility Treatment Center, Kermanshah, Iran. The research protocol was approved by the Research Ethics Committee of Lorestan University of Medical Sciences (code: IR.LUMS.REC.1405.321) and registered at the Iranian Registry of Clinical Trials (IRCT20114010716121N1).

### 2.2 Participants

The statistical population comprised all infertile couples referring to the Motazedi Infertility Treatment Center. The sample size was calculated a priori using G*Power software (version 3.1). The primary outcome for the sample size calculation was marital satisfaction, as measured by the ENRICH Marital Satisfaction Scale. Based on previous cognitive‑behavioral couple therapy (CBCT) studies conducted with distressed or infertile couples [1, 14]. we anticipated a large effect size (Cohen's d = 1.2) for the between‑group difference in post‑intervention marital satisfaction scores. This effect size is commonly reported for intensive couple‑based psychological interventions. For a two‑tailed independent‑samples t‑test comparing the intervention and control groups, with α = 0.05, power (1−β) = 0.80, and an allocation ratio of 1:1, the required total sample size was 20 couples (10 per group). To account for a potential 15% attrition rate, we aimed to recruit 24 couples (12 per group). Ultimately, using convenience sampling, 24 eligible couples who met the inclusion and exclusion criteria were enrolled and randomly assigned to the two groups.

**Inclusion criteria were:** (a) providing written informed consent, (b) continuity of the standard infertility treatment process, (c) absence of concurrent psychiatric disorders, (d) a minimum marital duration of 2 years, and (e) a baseline score below 100 on the ENRICH Marital Satisfaction Scale (indicating marital dissatisfaction) and below 40 on the Oxford Happiness Questionnaire (indicating low to moderate happiness). These threshold scores were chosen according to the cut‐off values established in previous similar studies [15, 16].

**Exclusion criteria were**: (a) presence of any acute physical illness that could hinder the psychotherapy process, (b) presence of psychotic symptoms (e.g., hallucinations or delusions), (c) diagnosis of personality disorders, and (d) absence from more than two therapy sessions. The evaluation was done by using Structured Clinical Interview for DSM Disorders (SCID). SCID is a semi‐structured clinical interview that provides diagnosis based on DSM‐IV.

### 2.3 Randomization and Blinding

Eligible couples were randomly assigned to either the intervention (*n* = 12 couples) or control (*n* = 12 couples) group using a simple randomization method (coin toss/random number table) performed by an independent researcher not involved in the therapeutic process. Due to the nature of the psychotherapeutic intervention, blinding of the participants and the therapist was not feasible. However, the data analyst and the statistician responsible for evaluating the outcomes were blinded to the group allocation (single‐blinded design).

### 2.4 Intervention

The experimental group received cognitive‐behavioral couples therapy (CBCT) based on the protocol developed by [17, 18] while the control group received only the routine infertility medical treatments (treatment as usual/TAU) without any psychological intervention. The CBCT intervention was delivered in 10 weekly sessions, each lasting 120 min. The sessions were conducted by a trained clinical psychologist who had received formal supervision in the CBCT protocol. A summary of the therapeutic content for each session is presented in Table 1.

**Table 1:** The summary of therapy sessions presented in the following table.

1th session	Establish a proper relationship with the couples, Statement of goals and regulations of the research and number of sessions and concluding a medical contract.
2nd session	Assessing and uniting therapy with couples so that each of them feels safe and accepted by the therapist and ensures that the therapist understands their goals and needs and will be able to help them.
3rd session	Individual and joint evaluation. Evaluate and determine the main objectives.
4th session	Teaching the effect of gender differences and couples' differences on communication and recommendations for understanding and observing differences.
5th session	Investigating Negative Patterns, Blaming and Blame, Threats to leave the relationship, Breaking the Marital Relationship, Identifying Life Stressing Factors.
6th session	Teaching the role of cognitive factors and cognitive errors in couples.
7th session	Creating plans for enjoyable activities, defining each other's positive traits, exchanging behaviors, enhancing individual activities.
8th session	Teaching communication skills, using verbs with my conscience, teaching listening skills, identifying interactive patterns, conflict resolution skills.
9th session	Teaching problem‐solving skills.
10th session	Summarizing, receiving feedbacks, concluding, and performing a post‐test

### 2.5 Follow‐up

All participants in both groups completed the outcome measures at three‐time points: baseline (pre‐test), immediately after the final session (post‐test), and at a 2‐month follow‐up to assess the stability of the treatment effects.

### 2.6 Outcome Measures

Two validated self‐report instruments were used to collect data at all three measurement points (baseline, post‐intervention, and 2‐month follow‐up). The primary and secondary outcomes were defined a priori as follows:

#### 2.6.1 Primary Outcome

The primary outcome of this trial was marital satisfaction, measured using the ENRICH Marital Satisfaction Scale. Marital satisfaction was selected as the primary outcome because it was the basis for our sample size calculation and represents the most direct target of couple‐based psychological interventions.

#### 2.6.2 Secondary Outcome

The secondary outcome was subjective happiness, as measured by the Oxford Happiness Questionnaire (OHQ). Happiness was considered a secondary outcome to evaluate the broader psychological well‐being effects of the intervention beyond the specific domain of marital functioning.

#### 2.6.3 Oxford Happiness Questionnaire (OHQ)

Developed by Argyle and Lu (1989), this 29‐item questionnaire measures subjective happiness. Each item is scored on a 4‐point Likert scale ranging from 0 to 3, yielding a total possible score between 0 and 87. The Persian version of the OHQ has demonstrated satisfactory psychometric properties; Alipour and Noorbala (1999) reported a Cronbach's alpha coefficient of 0.93 for the Iranian population [19].

#### 2.6.4 ENRICH Marital Satisfaction Scale

This scale consists of 47 items covering 11 subscales, including Idealistic Distortion, Marital Satisfaction, Personality Issues, Communication, Financial Management, Leisure Activities, Sexual Relationship, Children and Parenting, Family and Friends, Egalitarian Roles, and Conflict Resolution. Items are rated on a 5‐point Likert scale. The reliability of the total scale has been reported as 0.92 in the original version. In the Iranian context, Soleimani reported a Cronbach's alpha coefficient of 0.95 for the total scale, confirming its high reliability [20].

### 2.7 Statistical Analysis

Data analysis was performed using IBM SPSS Statistics for Windows, version 25.0 (IBM Corp., Armonk, NY, USA). Descriptive statistics (mean, standard deviation, frequency, and percentage) were calculated for demographic and baseline characteristics. To ensure the homogeneity of the groups at baseline, an Independent Samples t‐test was used for continuous variables and a Chi‐square test was used for categorical variables. The primary hypotheses regarding the intervention's effectiveness over time were evaluated using a 2 (Group: experimental vs. control) × 3 (Time: pre‐test, post‐test, follow‐up) repeated‐measures analysis of variance (ANOVA). In cases where the sphericity assumption was violated, the Greenhouse‐Geisser correction was applied. The significance level was set at a two‐tailed *p* ≤ 0.05 for all statistical tests.

## 3 Results

As illustrated in Figure 1, a total of 35 couples were assessed for eligibility. Of these, 11 couples were excluded for not meeting the inclusion criteria (*n* = 6), declining to participate (*n* = 3), or other reasons (*n* = 2). Finally, 24 eligible couples were randomized into the intervention (*n* = 12) and control (*n* = 12) groups. All couples in both groups received their allocated condition and completed the post‐test and 2‐month follow‐up assessments; no participants were lost to follow‐up or discontinued the study. Therefore, 12 couples in each group were included in the final analysis (Figure 1).

**Figure 1:**
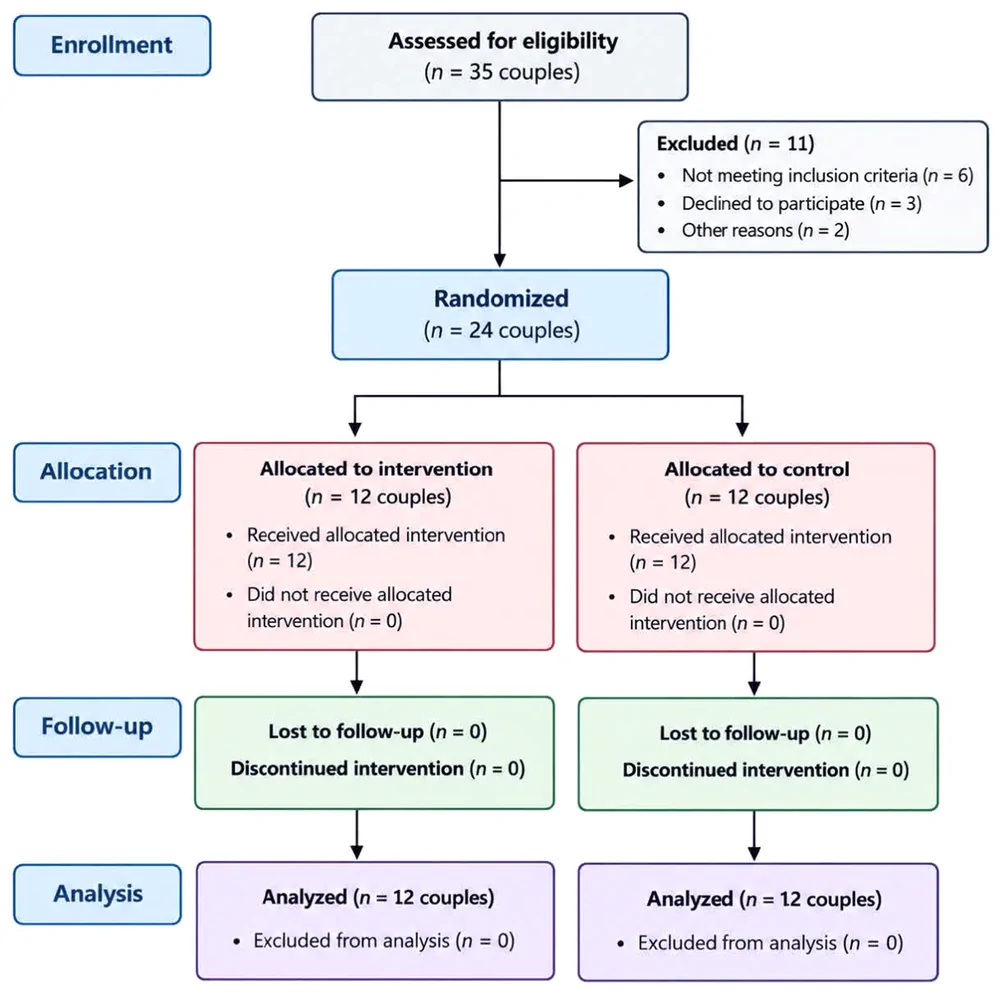
Flow diagram of participants through the study according to CONSORT guidelines.

Table 2 presents the baseline demographic and clinical characteristics of the participants across the intervention and control groups. A total of 24 couples (48 individuals) were included, with each group comprising 12 couples (12 females and 12 males). To ensure the initial equivalence of the groups, Fisher's exact test was conducted for all categorical variables due to the small sample size and expected cell counts below 5. No statistically significant differences were observed between the two groups for any of the assessed variables, including sex distribution (*p *= 0.999), level of education (*p *= 0.241), economic status (*p *= 0.825), history of referral to a general physician (*p *= 0.989) or a psychiatrist (*p *= 0.999), occupational status (*p *= 0.265), duration of infertility (*p *= 0.881), infertility etiologies (*p *= 0.904), and history of prior infertility treatments (*p *= 0.673). All p‐values were well above the significance threshold of 0.05, indicating that the two groups were homogeneous at baseline (Table 2).

**Table 2:** comparison of baseline demographic and clinical characteristics between the intervention and control groups.

Variables		Frequency (%)		*p*‐value
		Intervention (*N* = 12 couples)	Control (*N* = 12 couples)	
Sex	Female	12 (50)	12 (50)	0.999
	Male	12 (50)	12 (50)	
Level of education	Diploma	16 (59.3)	11 (40.7)	0.241
	Assistant	4 (30.8)	9 (69.2)	
	Masters	4 (50)	4 (50)	
Economic	Low	2 (16.7)	1 (8.3)	0.825
	Medium low	3 (25.0)	4 (33.3)	
	Medium	6 (50.0)	5 (41.7)	
	Medium high	1 (8.3)	2 (16.7)	
Refer to the doctor	Yes	3 (25.00)	2 (16.7)	.989
	No	9 (75.00)	10 (83.3)	
Refer to the psychiatrist	Yes	4 (33.3)	4 (33.3)	0.999
	No	8 (66.7)	8 (66.7)	
Job	Governmental	3 (33.3)	6 (66.7)	0.265
	Free	11 (64.7)	6 (35.3)	
	Unemployed	10 (45.5)	12 (54.5)	
Duration of infertility	2–3 year	4 (33.3)	4 (33.3)	0.881
	3–5 year	5 (41.7)	4 (33.3)	
	> 5 year	3 (25.00)	4 (33.3)	
Infertility etiologies	Male factor	5 (41.7)	4 (33.3)	0.904
	Female factor	5 (41.7)	6 (50.0)	
	Mixed factors	2 (16.6)	2 (16.7)	
History of prior infertility treatments	Yes	7 (58.3)	8 (66.7)	0.673
	No	5 (41.7)	4 (33.3)	

Table 3 presents the mean scores and standard deviations for happiness and marital satisfaction across the three assessment points (pre‐test, post‐test, and 2‐month follow‐up) for both the intervention and control groups, along with the corresponding between‐ and within‐group p‐values. With respect to happiness, the two groups were statistically comparable at baseline (*p *= 0.83); however, at the post‐test and follow‐up stages, the intervention group exhibited significantly higher happiness scores compared to the control group (*p *< 0.001 for both time points). Moreover, within‐group repeated measures analyses revealed a substantial and statistically significant improvement in happiness scores across time exclusively for the intervention group (*p *< 0.001), whereas the control group demonstrated no notable fluctuation (*p *= 0.92). (Table 3, Figure 2).

**Table 3:** descriptive statistics (Mean ± SD) and between‐group comparisons for happiness and marital satisfaction at pre‐test, post‐test, and two‐month follow‐up.

		Mean ± SD		*p*‐value
		Intervention	Control	
Happiness	Pre‐test	64.79 ± 7.92	65.29 ± 8.85	0.83
	Post‐test	76.20 ± 7.49	63.75 ± 12.57	*p* < 0.001
	Follow‐up	78.62 ± 10.19	64.33 ± 10.25	*p* < 0.001
	*p*‐value	*p* < 0.001	0.92	—
Marital satisfaction	Pre‐test	145.54 ± 17.37	156.79 ± 21.66	0.053
	Post‐test	174.62 ± 26.44	155.62 ± 25.21	0.01
	Follow‐up	177.25 ± 21.37	156.08 ± 23.12	0.002
	*p*‐value	*p* < 0.001	0.85	—

**Figure 2:**
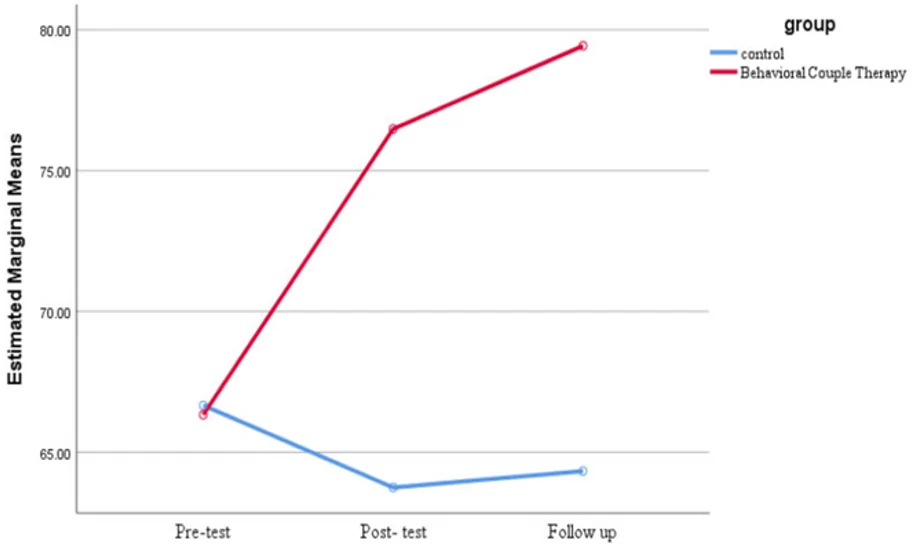
Graph of changes in happiness scores between experimental and control groups in three stages of pre‐test, post‐test and follow‐up.

Regarding marital satisfaction, although the baseline difference between the groups approached significance (*p *= 0.053), the groups were considered essentially homogeneous at pre‐test. Following the intervention, the CBCT group scored significantly higher than the control group at both the post‐test (*p *= 0.01) and the follow‐up assessment (*p *= 0.002). Furthermore, only the intervention group displayed a significant upward trend in marital satisfaction over the course of the study (*p *< 0.001), while the control group remained stable over time (*p *= 0.85). (Table 3, Figure 3).

**Figure 3:**
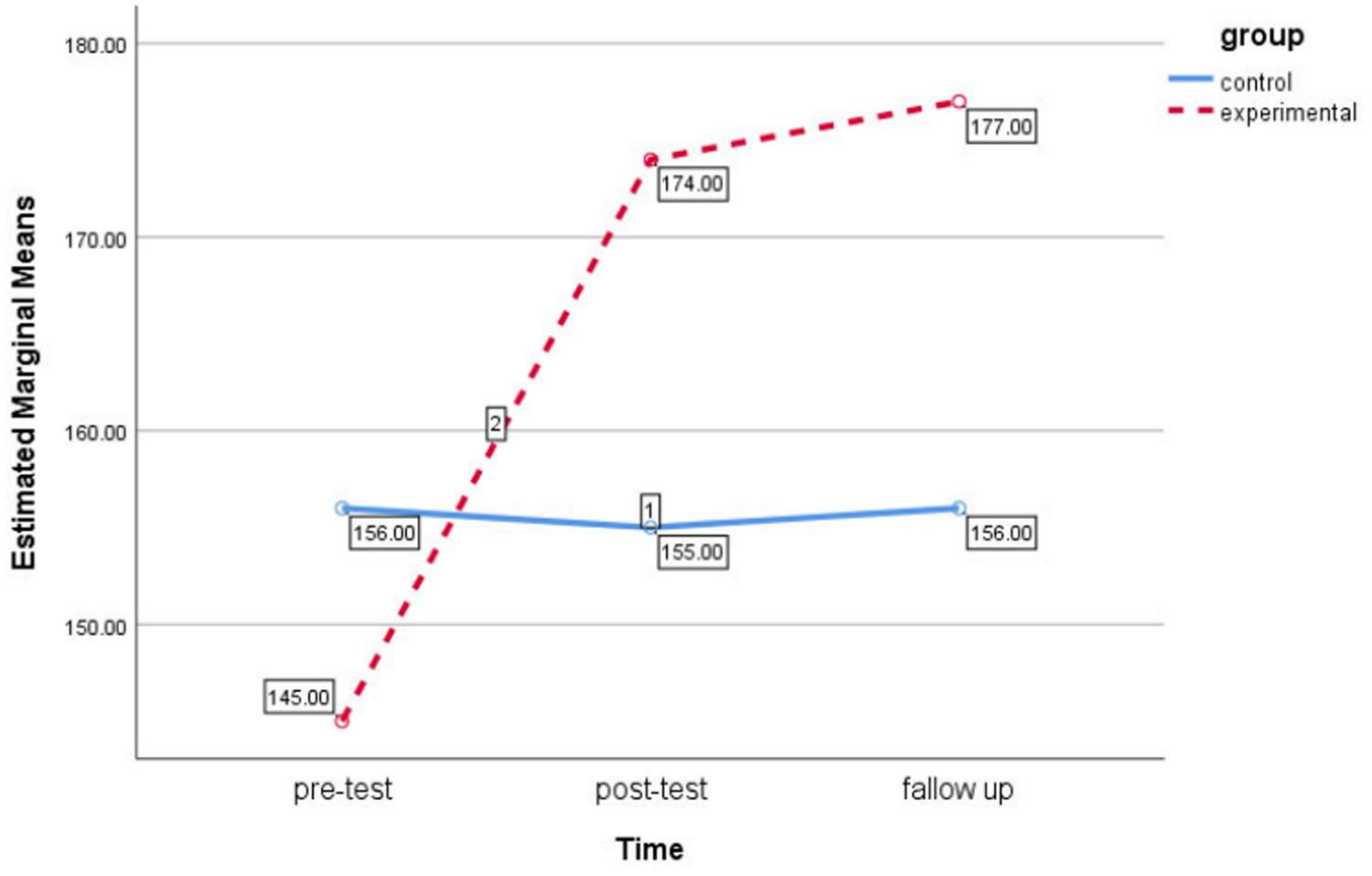
Graph of changes in marital satisfaction scores between experimental and control groups in three stages of pre‐test, post‐test and follow‐up.

To evaluate the differential trajectories of change over time between the intervention and control groups, a 2 (Group) × 3 (Time) repeated‐measures analysis of variance (ANOVA) was conducted for happiness and marital satisfaction, with the results summarized in Table 4. For happiness, the analysis revealed a significant main effect of time, *F* (2, 86) = 5.87, *p* = 0.004, partial η^2^ = 0.120, indicating an overall change across the three assessment points. More importantly, the time × group interaction was highly significant, *F* (2, 86) = 13.48, *p* < 0.001, partial η^2^ = 0.239, observed power = 0.997, demonstrating that the pattern of change in happiness scores over time differed significantly between the two groups. Similarly, for marital satisfaction, a significant main effect of time was observed, *F* (2, 86) = 10.70, *p* < 0.001, partial η^2^ = 0.199, alongside a substantial and highly significant time × group interaction, *F* (2, 86) = 16.36, *p* < 0.001, partial η^2^ = 0.276, observed power = 1.000. Collectively, these findings confirm that the Cognitive‐Behavioral Couples Therapy intervention produced a significantly distinct upward trajectory of improvement across time for both primary outcomes compared to the control group. The large effect sizes (partial η^2^ ranging from 0.239 to 0.276) indicate that a substantial proportion of the variance in outcome changes over time is attributable to the group allocation, while the observed power values (all exceeding 0.86) confirm that the study was adequately powered to detect these effects, thereby reinforcing the robustness and clinical significance of the intervention's impact (Table 4).

**Table 4:** Repeated‑Measures ANOVA for Primary Outcomes: Main and Interaction Effects.

Variable	Source	Type III sum of squares	df	Mean square	F	*p*‐value	Partial Eta squared	Observed power^a^
Happiness	Time	674.00	2	337.00	5.87	0.004	0.120	0.864
	Time * group	1546.36	2	773.18	13.48	*p* < 0.001	0.239	0.997
	Error (factor1)	4932.11	86	57.35				
Marital satisfaction	Time	6264.30	2	3132.15	10.70	*p* < 0.001	0.199	0.988
	Time * group	9578.02	2	4789.01	16.36	*p* < 0.001	0.276	1.000
	Error (factor1)	25159.96	86	292.55				

## 4 Discussion

The aim of the present study was to investigate the effectiveness of cognitive behavioral couple therapy on increasing happiness and marital satisfaction among infertile couples. The results showed that cognitive behavioral couple therapy significantly increased happiness and marital satisfaction among infertile couples. That is, the couples in the experimental group had a higher level of happiness and marital satisfaction after a 2‐month follow‐up compared to the control group. In other words, the scores of post‐tests were different in the two groups, indicating the effects of education and treatment in the experimental group.

A recent systematic review and meta‐analysis by Alirezaei et al. (2022), which included 13 randomized clinical trials comprising 230 infertile women and 512 infertile couples, firmly established that counseling and psychological interventions significantly improve marital and sexual satisfaction among infertile couples [21]. Furthermore, a 2023 systematic review by Yahyavi Koochaksaraei et al. identified cognitive behavioral therapy (CBT) and mind‐body interventions as the most frequently and effectively employed approaches for promoting mental health dimensions in infertile women [22].

More recently, Yamanaka‐Altenstein (2023) introduced a demand‐oriented cognitive‐behavioral couple therapy concept for couples with infertility (FERTIFIT), reporting that women showed improved psychological and infertility‐related distress, applied more resources fostering subjective well‐being, active coping and emotion regulation, and both women and men reported high satisfaction with the intervention. A 2024 study demonstrated that cognitive‐behavioral group therapy leads to increased marital satisfaction in infertile women applying for IVF [23].

Infertile couples experience more negative communication in their common life and the level of empathy decreases in their relationships. In a research, Moura et al. stated that infertile couples were less involved in solving their problems than other couples. They believed that marital satisfaction was lower in infertile couples than in other couples [24]. The results of our previous research showed that the level of mental health, happiness and marital satisfaction were higher in fertile women than infertile women; inferiority feelings and marital conflict were stronger in infertile women [25]. In a study, Gardi [5] and Barani [26] showed that marital dissatisfaction was higher in infertile couples and their satisfaction went higher after psychological intervention. Likewise, Najafi et al. believed that emotion‐based couple therapy was effective to increase marital adjustment [27]. Khaleghkhah showed that couple therapy increased the mental health and marital satisfaction of couples [28]. Nazari et al. showed that behavioral therapy was more effective than cognitive therapy [29]. Asazawa et al. showed significantly improved parts of the QoL of men who were infertile due to various causes [3].

Supporting these findings, a 2024 study by Banaha et al. demonstrated that group psychosexual training significantly improved marital adjustment and its subscales, including marital consensus and satisfaction, among infertile women in a randomized controlled trial. Furthermore, recent evidence suggests that couple collaboration strategies are associated with higher well‐being during infertility, indicating that mental health programs for infertile couples should incorporate strategies based on couple collaboration [30].

Happiness is not a personal feeling, but it is an achievement of things that are precious from people's perspective [8]. Values such as self‐esteem and social acceptability show a high correlation with happiness. Accordingly, the greater the social acceptability, the more their happiness will be. In a research, Zare showed that infertile women had lower self‐esteem than fertile ones [31]. The results of this study were in line with the results of the research by Almasi. He suggested that infertile women had a lower level of happiness than other people. In his research, he showed that there was a strong and positive correlation between life satisfaction and happiness [8]. The results of this study were consistent with our previous study showing that general health of infertile women could be affected by happiness and inferiority feeling [32]. The results of the studies done by Goli et al. [26] and Moura‐Ramos [18] indicated that the degree of willingness to bear a child and the infertile people's mental stress had an effect on their marital satisfaction.

In explaining these findings, it can be noted that the contributing factors of conflicts and of reducing satisfaction and happiness in life are issues that make couples unable to communicate properly. This communication can be improved by training. Communication plays a key role in people's common/marital life and it should be paid particular attention. Therefore, the couple therapy approach with 10 sessions can be useful as it is a branch of marital therapies on the one hand and it places priority on communication on the other hand. This type of therapy focuses on communicative compatibility/disagreements and encourages people to correct them. A 2025 systematic review by Salarfard et al. confirmed that various counselling approaches, including self‐regulatory couple therapy, emotion‐focused couple therapy, and integrated behavioral approaches, effectively improve marital intimacy among women and couples experiencing infertility [33].

The review of literature showed that educational intervention in the field of couple therapy would improve communication and marital satisfaction. With the increase of happiness in the family, couple's relationships will improve. The communicative problems with any source can be improved by appropriate training in order to acquire proper communicative skills that are fruitful of improving marital relationship. So, these therapies can be used for infertile couples in infertility clinics. Contemporary literature increasingly supports the integration of assisted reproductive technology with psychosocial support systems, including cognitive‐behavioral therapy, mindfulness interventions, and patient‐centered counseling. As CBT, complemented by third‐wave elements, is now considered a recommended form of support for couples facing infertility issues, infertility clinics should consider incorporating structured couple therapy programs into their standard care protocols to address the psychological and relational needs of infertile couples comprehensively.

This study possesses several methodological and conceptual strengths that distinguish it from previous investigations. Foremost among these are the rigorous randomized controlled trial (RCT) design, the inclusion of a two‑month follow‑up period to assess the sustainability of treatment effects, and the use of validated, culturally‑adapted instruments with high psychometric reliability in the Iranian population. Conceptually, while the majority of prior research has focused on alleviating negative psychological states—such as depression, anxiety, or marital conflict—among infertile couples, the present study is novel in its concurrent targeting of two positive psychological constructs, as primary outcomes. This dual‑focus approach provides a more comprehensive and strengths‑oriented perspective on couple functioning. Furthermore, to the best of our knowledge, this is one of the first randomized trials to implement a structured, 10‑session cognitive‑behavioral couple therapy (CBCT) protocol specifically adapted for infertile couples within an Iranian clinical context, where cultural and religious values profoundly shape marital expectations, communication patterns, and coping strategies. By demonstrating that CBCT can significantly enhance both relational and individual well‑being in this specific population, our findings add culturally‑relevant evidence to the global infertility literature and offer a practical, evidence‑based framework for integrating psychological support into routine reproductive health services in Iran.

### 4.1 Limitations

Despite the robust findings, several limitations should be considered when interpreting the results of this study. First, the relatively small sample size (24 couples) and the use of convenience sampling from a single infertility center in Kermanshah limit the external validity and generalizability of the findings to broader populations of infertile couples across diverse sociodemographic and clinical settings. Second, the psychotherapeutic nature of the intervention precluded the blinding of participants and the therapist, which may have introduced performance and detection biases; although the data analyst remained blinded to group allocation, the lack of double‐blinding remains a methodological constraint. Third, reliance exclusively on self‐report questionnaires (OHQ and ENRICH) may have exposed the data to social desirability and recall biases, potentially affecting the accuracy of the reported outcomes. Fourth, the follow‐up period was restricted to 2 months, which limits our ability to assess the long‐term sustainability and durability of the therapeutic gains over an extended timeframe. Future multicenter trials with larger sample sizes, longer follow‐up periods, and the inclusion of objective behavioral measures are warranted to further validate and extend the present findings. Although we systematically collected data on infertility duration, etiologies, and history of prior treatments as baseline clinical characteristics, the present study did not specifically assess or control for the type, stage, intensity, or cycle number of the ongoing medical treatments (e.g., IUI vs. IVF/ICSI, first vs. repeated cycles) that participants were concurrently receiving during the psychological intervention. These medical parameters may influence stress levels, treatment expectations, and overall psychological well‐being, potentially moderating the effectiveness of the CBCT intervention. Future studies are encouraged to incorporate detailed documentation of current medical treatment protocols and to examine their interactions with psychological outcomes.

## 5 Conclusion

This treatment approach can significant increase in the happiness and marital satisfaction of infertile couples. Participants were satisfied with the sessions. The current results of the research can be a practical guide for effective communication methods between couples and reduce their inconsistencies. Cognitive Behavioral Couples Therapy Program can be used to make infertility treatment in infertility clinics.

### 5.1 Future Perspectives

Future research should prioritize large‐scale, multi‐center trials with extended follow‐up to confirm the generalizability of our findings. Additionally, stratifying participants by the type and stage of concurrent medical treatments (e.g., IUI vs. IVF) could help identify moderators of treatment response. Comparative studies against other interventions (e.g., mindfulness‐based or emotion‐focused therapies) and the exploration of online delivery formats would further enhance the clinical applicability of CBCT for infertile couples in Iran.

## Author Contributions

**Malihe Gholamzade Deharab:** investigation, writing – original draft, writing – review and editing, methodology. **Jamile Shahverdi:** investigation, writing – original draft, writing – review and editing, methodology. **Elham Goodarzi:** investigation, writing – original draft, writing – review and editing, methodology. **Azam Ardeh:** writing – original draft, writing – review and editing. **Seyed Mojtaba Ahmadi:** writing – original draft, writing – review and editing.

## Funding

The authors have nothing to repor.

## Ethics Statement

This research was approved by the Ethics Committee of Lorestan University of Medical Science) IR.LUMS.REC.1405.321 (and performed in accordance with the Declaration of Helsinki. All participants provided their informed consent prior to beginning any study activities.

## Conflicts of Interest

The authors declare no conflicts of interest.

## Transparency Statement

The corresponding author, Jamile Shahverdi and Elham Goodarzi, affirms that this manuscript is an honest, accurate, and transparent account of the study being reported, that no important aspects of the study have been omitted; and that any discrepancies from the study as planned (and, if relevant, registered) have been explained.

## Data Availability

The data that support the findings of this study are available from the corresponding author upon reasonable request.
